# Assembly of Surface-Mounted Devices on Flexible Substrates by Isotropic Conductive Adhesive and Solder and Lifetime Characterization

**DOI:** 10.3390/mi13081240

**Published:** 2022-08-01

**Authors:** Rafat Saleh, Sophie Schütt, Maximilian Barth, Thassilo Lang, Wolfgang Eberhardt, André Zimmermann

**Affiliations:** 1 Hahn-Schickard, Allmandring 9b, 70569 Stuttgart, Germany; maximilian.barth@hahn-schickard.de (M.B.); wolfgang.eberhardt@hahn-schickard.de (W.E.); zimmermann@ifm.uni-stuttgart.de (A.Z.); 2 Institute for Micro Integration (IFM), University of Stuttgart, Allmandring 9B, 70569 Stuttgart, Germany; st152565@stud.uni-stuttgart.de (S.S.); thassilo.lang@gmail.com (T.L.)

**Keywords:** bending, conductive adhesive, dynamic bending, flexible electronics, flexible circuits, passive components, roll to flex, shear strength, SMDs, system-in-foil, reliability

## Abstract

The assembly of passive components on flexible electronics is essential for the functionalization of circuits. For this purpose, adhesive bonding technology by isotropic conductive adhesive (ICA) is increasingly used in addition to soldering processes. Nevertheless, a comparative study, especially for bending characterization, is not available. In this paper, soldering and conductive adhesive bonding of 0603 and 0402 components on flexible polyimide substrates is compared using the design of experiments methods (DoE), considering failure for shear strength and bending behavior. Various solder pastes and conductive adhesives are used. Process variation also includes curing and soldering profiles, respectively, amount of adhesive, and final surface metallization. Samples created with conductive adhesive H20E, a large amount of adhesive, and a faster curing profile could achieve the highest shear strength. In the bending characterization using adhesive bonding, samples on immersion silver surface finish withstood more cycles to failure than samples on bare copper surface. In comparison, the samples soldered to bare copper surface finish withstood more cycles to failure than the soldered samples on immersion silver surface finish.

## 1. Introduction

System-in-foil (SiF) opens new possibilities for applications, such as wearables for monitoring human health, implantable electronics for imaging and diagnostics, robotics, and digitalization [1]. Depending on the substrates used, these systems are flexible and even stretchable, and they allow a higher packaging density [2,3]. In addition, such assemblies can be manufactured as large-area devices and allow heterogeneous integration of different components. These features make such systems very attractive for the Internet of Things, consumer electronics, healthcare, automotive, and, last, but not least, aerospace [4,5,6,7,8]. For the realization of flexible electronics, including SiF, both silicon chips and passive components such as resistors are required [9]. As passive components mostly, surface-mounted devices (SMD) are integrated to provide circuit functionality [10]. The circuit substrates used for flexible systems are predominantly polyethylene terephthalate (PET), polyethylene naphthalate (PEN), polycarbonate (PC), polyethersulfone (PES), liquid crystal polymer (LCP), and polyimide (PI) [11,12]. Depending on the circuit substrate used, there are different requirements for the assembly process of the SMD. Especially, the thermal and mechanical properties of the substrates are essential. Common assembly technologies for SMDs on flexible and hybrid electronics are soldering and methods using isotropic conductive adhesives (ICA) [13,14]. Although soldering provides higher mechanical reliability compared to conductive adhesive bonding, sufficient reliability with conductive adhesive bonding can be achieved by process and material optimization [10,15]. Even though the assembly of SMDs on foils is an industry standard, there is little literature available on reliability, especially under dynamic bending conditions [16,17,18,19,20]. Furthermore, there is a lack of knowledge about the comparison of different assembly and packaging technologies, including soldering and conductive adhesive bonding, and their influence on the bending characterization of SiF.

This paper investigates the assembly process of SMD on flexible polyimide substrates by soldering and conductive adhesive bonding and its influence on reliability by using shear and dynamic bending tests.

## 2. Materials and Methods

### 2.1. Shear Strength Characterization

Different technologies for the assembly of SMDs on flexible polyimide substrates are investigated. For this purpose, a variation of materials and process parameters is carried out for a subsequent shear test. The aim of the shear test is to identify the relevant parameters influencing the shear strength of the SMDs.

**Figure 1:**
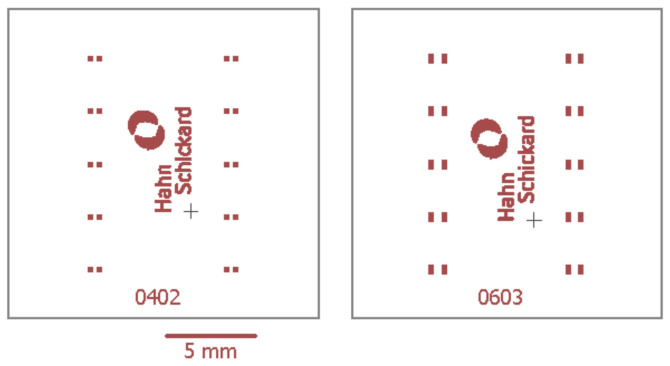
Circuit layout for shear tests of 0402 and 0603 SMDs (surface mounted devices).

Copper-clad polyimide foils with rolled-annealed copper (MC-18-25-00-CEM-Z-0300-0500, Holders Technology, Kirchheimbolanden, Germany) with a polyimide and copper thickness of 25 µm and 18 µm, respectively, are used as a substrate. The copper-clad foils are structured and etched using lithographic processes. To perform shear tests, the structured foils are laminated on copper-clad PCBs (FR4 basis material (PCB), Lerrox, Aalen-Ebnat, Germany). Figure 1 shows the schematic circuit layout for the shear test of 0603 and 0402 SMD resistors. The contact pads are 0.9 mm × 0.6 mm for 0603 SMDs and 0.6 mm × 0.5 mm for 0402 SMDs. Both layouts feature ten mounting positions for the corresponding SMD size.

The assembly technologies continuous furnace soldering, vapor phase soldering, and conductive adhesive bonding are investigated comparatively. Two different adhesives are investigated, Loctite 3880 (Loctite Ablestik 3880, Henkel AG & Co. KGaA, Duesseldorf, Germany) as adhesive A, and H20E (EPO-TEK H20E, Epoxy Technologies, Inc., Billerica, MA, USA) as adhesive B. Both adhesives are based on epoxy resin filled with silver particles. Adhesive A is available as a single component. Adhesive B, in contrast, consists of two components, but it is supplied premixed by the manufacturer. In addition to these adhesives, solder pastes based on different solder alloys are also investigated. SAC solder (F 169 SA40C5-86D30, Heraeus Holding GmbH, Hanau, Germany), solder A, is selected as a standard solder material according to the widespread use on printed circuit boards. However, for temperature-sensitive assemblies, solder material with lower melting point can be applied [9]. Therefore, SnBi solder (S42D500A5, Nordson EFD, East Providence, RI, USA), solder B, is used. The addition of silver to the solder material has a positive effect on the brittleness [7]. For this reason, SnBiAg (DP 5600, Interflux Electronics, Gent, Belgium), solder C, is also used. Table 1 shows the test plan for the subsequent shear test in the two categories of soldering and conductive adhesive bonding.

**Figure 2:**
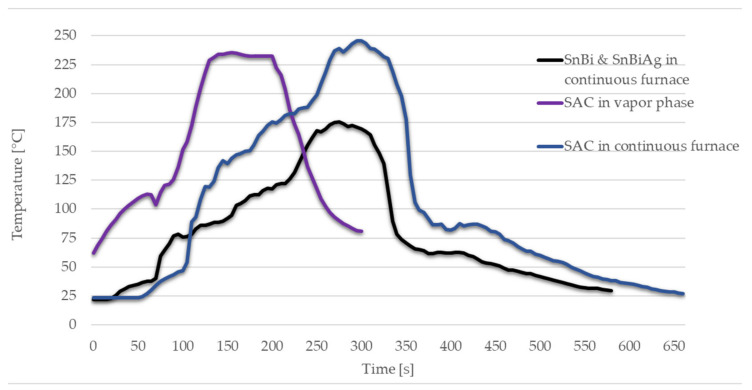
Soldering profiles in continuous furnace and in vapor phase.

The adhesive is stamped to the pads on the substrate with a stamp manufactured by 3D printing. To vary the amount of adhesive, the adhesive is applied on metal plates with two levels of adhesive reservoir depth (100 µm and 150 µm). The stamping process ensures that a uniform amount of adhesive is placed under the component. The adhesive quantity and volume depend on several factors, such as wetting on the substrate, viscosity of the adhesive, and placement process. The curing profiles are selected based on the data sheets of the adhesives [21,22]. For soldering, the solder is applied directly on the pad by a pressure-controlled manual dispenser (JB113N, Fisnar Inc., Germantown, WI, USA). In the first experiment, solder A is soldered both in the vapor phase (SLC 500, IBL-Löttechnik GmbH, Königsbrunn, Deutschland) and in the continuous furnace (SMT XXS, SMT Maschinen- und Vertriebs GmbH & Co. KG, Wertheim, Deutschland). Solder B and C are soldered in the continuous furnace. Figure 2 shows the used solder profiles.

After curing or soldering of the samples, a shear test is performed on a bond tester (Nordson DAGE 4000Plus, Nordson EFD, USA). The shear speed is set to 100 µm/s.

### 2.2. Dynamic Bending Characterization

**Figure 3:**
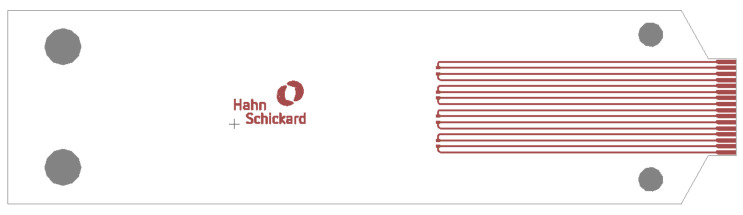
Circuit layout for dynamic bending test of foils with assembled 0402 and 0603 SMDs.

Figure 3 shows the circuit layout used for the bending tests. The substrate materials, as well as the pad geometries, are the same as described in Section 2.1.

Based on the results of the shear tests, the process parameters, adhesive reservoir depth 150 µm, curing profile 150 °C/12 min, and continuous furnace soldering, are selected for the bending tests. Table 2 shows the factors and levels used for DoE of dynamic bending tests.

**Figure 4:**
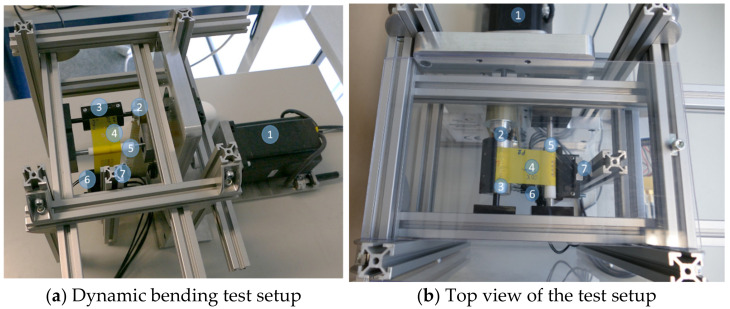
Configuration of the dynamic bending test setup. A motor (1) drives a shaft (2) via a belt. A winding roller (3) is mounted over the shaft. The sample foil (4) to be tested is fixed onto the winding roller. As the motor rotates back and forth, the bending foil is slid over an exchangeable roller (5). This roller determines the bending radius. The foil at the distal end is fixed to the readout electronics (6) to simultaneously monitor the resistances during bending. The electronics slide over a linear guide (7). The weight of the electronics is about 200 g and thus applies a tensile stress of about 2 N to the sample foil during testing.

After assembly, the samples are fixed and bent on the dynamic bending test setup (Figure 4). In preliminary tests, the samples are bent to different radii. Only at a bending radius of 5 mm could failures be generated. For this reason, a bending radius of 5 mm is applied here.

The DOE of the shear and bending tests is created and analyzed using the methods of the statistical analysis program Minitab (Minitab 18, Minitab, LLC, State College, PA, USA).

## 3. Results

### 3.1. Shear Strength

**Figure 5:**
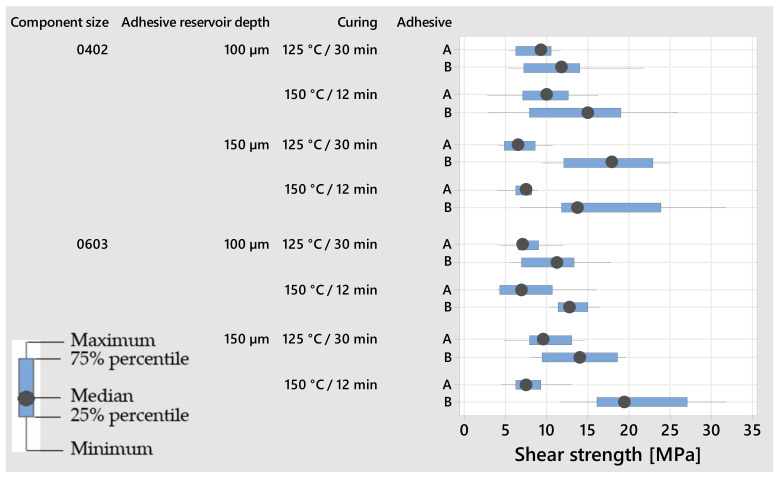
Shear strength of the conductively bonded SMDs on foil for the process variations.

To determine the shear areas, after the shear test of the SMD for 20 samples, the sheared area is individually measured by microscopy for each of the processed variations. The shear areas are 0.534 and 0.264 N/mm^2^ for 0603 and 0402, respectively. These determined shear areas are used to calculate the shear strength. Using the measured shear forces *F* and the determined shear areas *A*, the shear strength *τ* was calculated according to the formula τ=F/A. Figure 5 demonstrates the shear strength of the conductively bonded SMDs on polyimide foil based on ten replications each.

Based on the results for 0603, the highest shear strength could be achieved with the factors adhesive B, a large adhesive reservoir depth, and faster curing. In comparison, the highest shear strength values for 0402 could be obtained with the factors adhesive B, a large adhesive reservoir depth, and slow curing. To look more closely at the influence of the adhesive reservoir depth and curing profile, the results of the respective groups are subjected to a Student’s *t*-test (*t*-test) with a significance level of 5%. Using the *t*-test, the statistical significance of the differences between the means of two groups at the selected risk level is examined. There is no significant difference in the shear strengths obtained with the different adhesive reservoir depth and curing profiles when considering the *t*-test.

**Figure 6:**
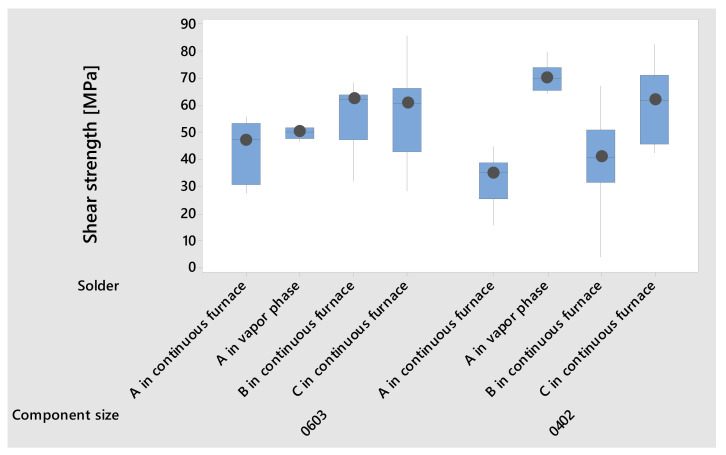
Shear strength of the soldered SMDs on the polyimide foil for the process variation.

For the soldered SMDs, higher shear strength could be achieved for the component size 0603 with the factors solder B and C, respectively, and continuous furnace soldering. However, the factors solder C in continuous furnace soldering and solder A in vapor phase soldering provided higher shear strength for 0402 (Figure 6).

**Figure 7:**
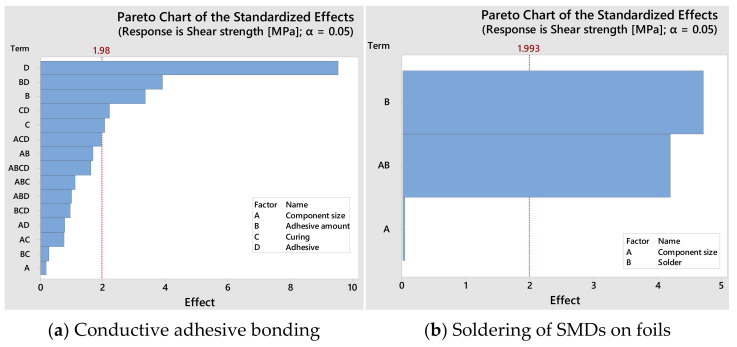
Pareto chart of the effects of the parameters influencing the shear strength.

**Figure 8:**
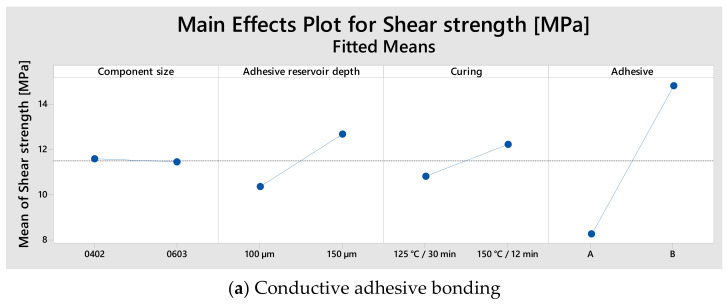

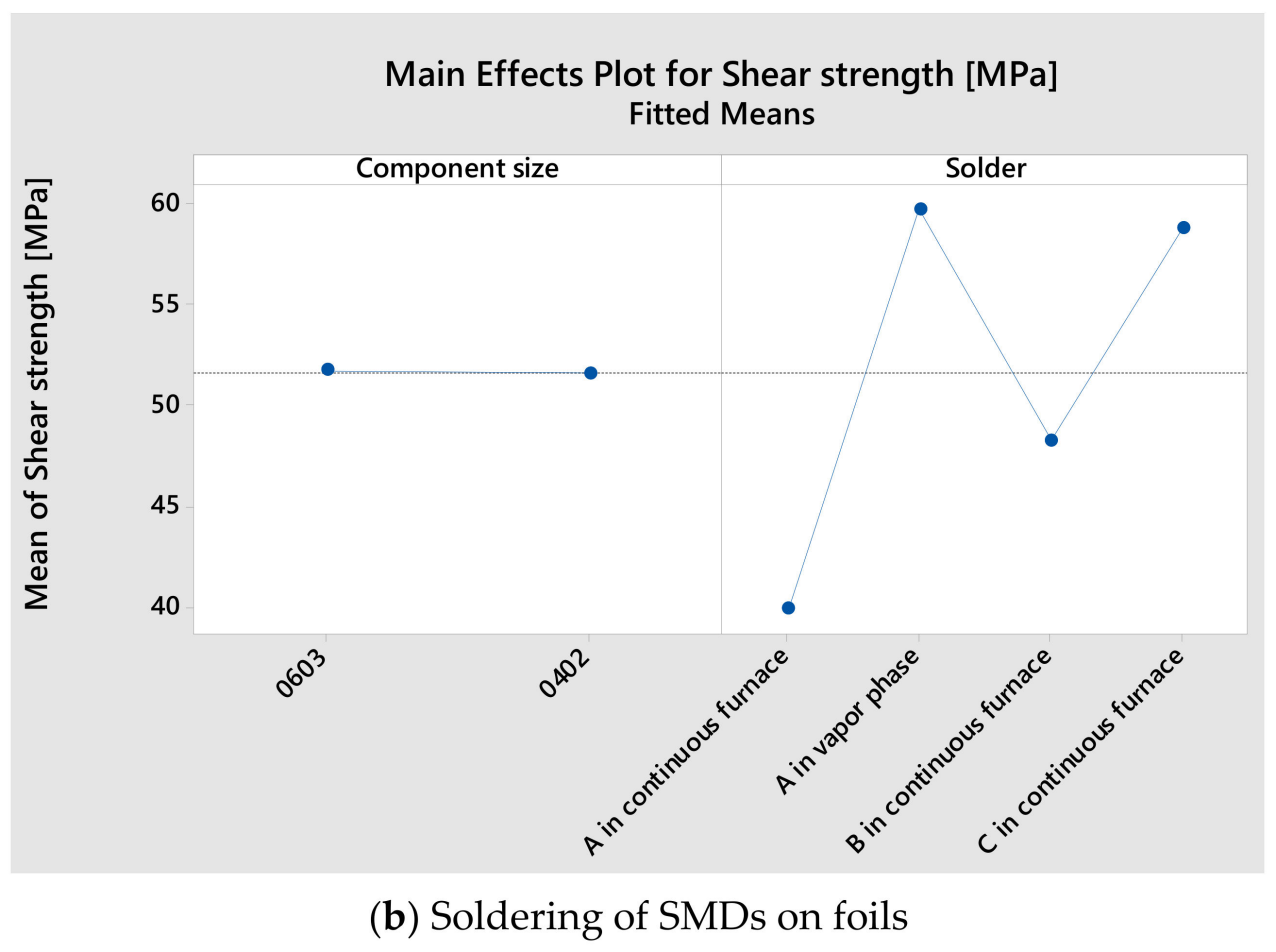
Main effect plots of the parameters influencing the shear strength.

Figure 7 and Figure 8 demonstrate the effect of the factors on the shear strength during soldering and conductive adhesive bonding.

Considering the determined shear strength during soldering, it was found that the 0603 soldered samples with solder B and C in continuous furnace soldering achieved higher shear strength values. This observation was verified utilizing the *t*-test. The achieved shear strength values were not significantly different for both groups (*t*-value = 0.05, DF = 15, *p*-value = 0.964). For the soldered 0402 samples, there was also no significant difference between the soldered samples with solder C in continuous furnace soldering and solder A in vapor phase soldering (*t*-value = −2.16, DF = 11, *p*-value = 0.054). In summary, the achieved shear strength values for soldering in all levels were higher than those for conductive adhesive bonding.

### 3.2. Bending Characterization

For each configuration of factors and levels shown in Table 2, eight samples are used for bending characterization. Four samples are bent simultaneously on the bending setup and the electrical resistances are recorded simultaneously during bending by a 5 mm bending radius. Bending is performed for 50,000 bending cycles. A resistance change of 20% from the initial resistance is defined as the failure condition. Some samples fail earlier, while the others remain intact until the end of the test. For this reason, the number of bending cycles to failure is divided into four categories for each configuration: failures between 1500 and 15,000 cycles, failures between 15,000 and 30,000 cycles, failures between 30,000 and 50,000 cycles, and intact samples at the end of the bending test.

**Figure 9:**
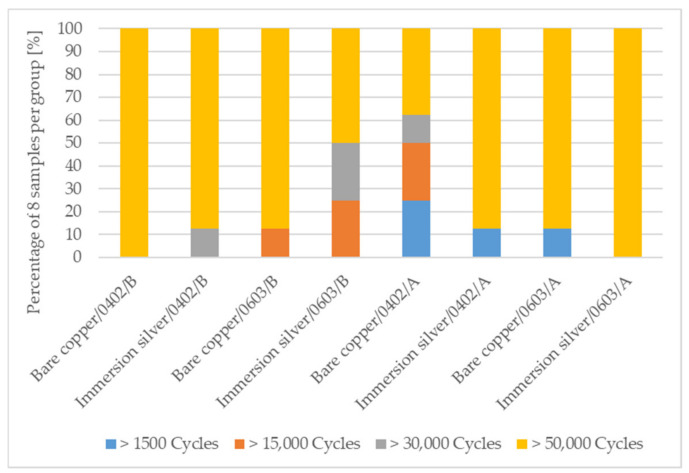
Achieved dynamic bending cycles to failure in categories for the different process variation for conductively bonded SMDs on foil.

Figure 9 shows the results of the bending test for conductively bonded samples for the individual factors in different categories. Early failures could be detected only for samples which were conductively bonded with adhesive A. One trend to note is that the samples conductively bonded with adhesive A on immersion silver surface finish withstood more cycles to failure than the samples on bare copper surface. Adhesive B seems to be more suited for conductive adhesive bonding on bare copper surface.

**Figure 10:**
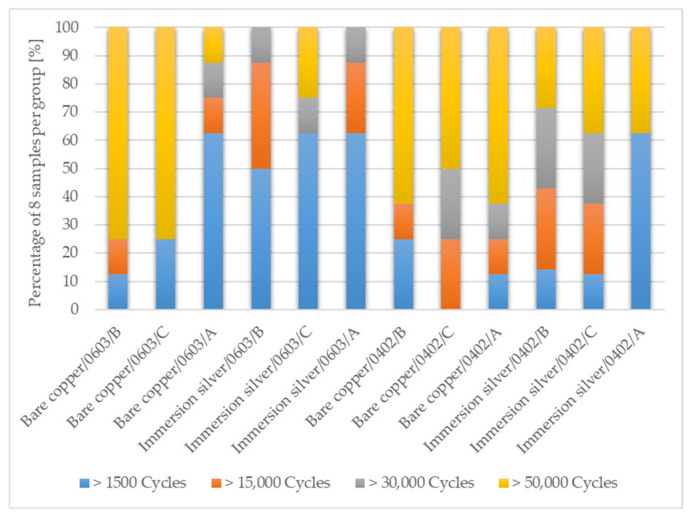
Achieved dynamic bending cycles to failure in categories for the different process variation for soldered SMDs on foil.

Figure 10 shows the results of the bending test for soldered samples for the individual factors in different categories. Early failures could be detected in all sample configurations. There is a trend that samples soldered with solder B and C on bare copper surface withstood more cycles of the bending test than samples soldered with solder A. Furthermore, there is a trend that samples soldered on immersion silver surface finish withstood fewer cycles to failure than the soldered samples on bare copper surface.

**Figure 11:**
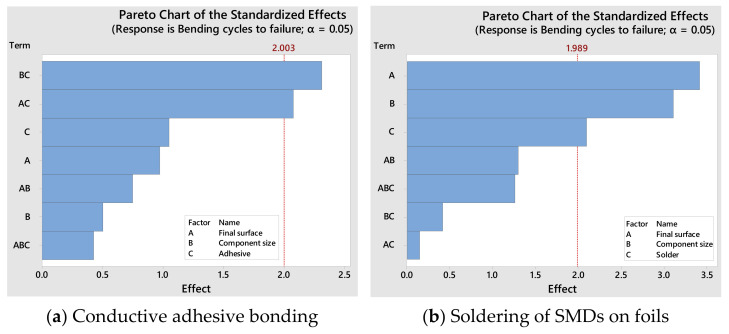
Pareto charts of the effects of the parameters influencing the bending cycles to failure.

Figure 11 shows the effect of the factors on the bending behavior of the samples and includes the number of cycles to failure for soldering and conductive adhesive bonding.

**Figure 12:**
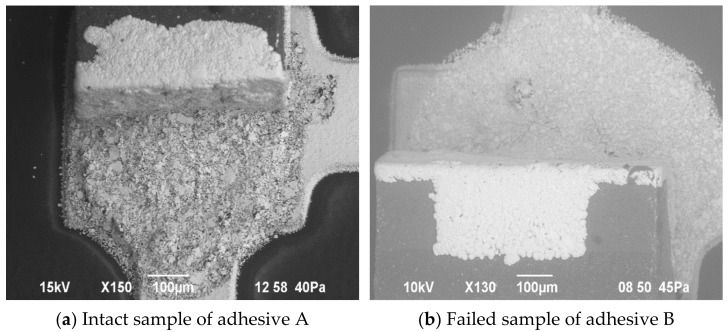
SEM pictures of conductively adhesive bonded SMDs after bending characterization.

**Figure 13:**
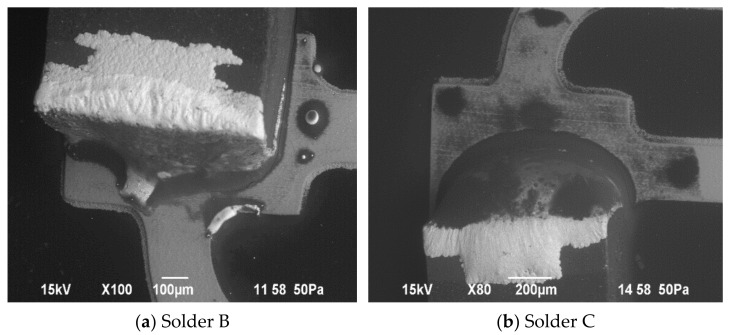
SEM pictures of failed soldered SMDs after bending characterization.

The following Figure 12 and Figure 13 show the assembled SMDs on flexible foil with both conductive adhesive bonding and soldering. On close inspection of the interface SMDs/solder or adhesive, the cracks are visible. The assemblies were investigated by scanning electron microscopy (JSM-6490LV, JEOL USA Inc., Peabody, MA, USA). Thereby, the images contain intact and failed samples after bend testing.

## 4. Discussion

Based on the shear strength results, adhesive B results in a higher shear strength for both component sizes, 0603 and 0402. In general, it can be derived from different variants that faster curing of the adhesive leads to increase in shear strength. Moreover, Zhang et al. [23] found that increasing the curing temperature led to the enhancement of shear strength.

Reducing the amount of adhesive B decreased the shear strength. However, increasing the amount of adhesive B did not contribute to any increase in shear strength.

Compared to conductive adhesive bonding, the highest shear strength in soldering of both component sizes is achieved with solder C. The shear strength values of the solder A obtained by vapor phase soldering are higher than those of the other solders for 0402, but for component size 0603, the shear strength values obtained by vapor phase and continuous furnace soldering are similar. For this reason, only a continuous furnace is used for the main tests.

In the bending test, the final surface finish on the substrate has the greatest effect on the number of cycles to failure. The advantage of the investigated surfaces is the low manufacturing cost compared to coatings such as ENIG [24,25].

The immersion silver surface finish provides a better interface to the silver flakes in the conductive adhesive, leading to increased reliability. There is also a tendency for adhesive A to show better bending behavior compared to adhesive B. One reason for this could be the slightly increased stiffness of adhesive B.

Bare copper tracks usually show very good solderability, but they have a tendency for surface oxidation during storage which can reduce solderability. Although the immersion silver coating offers improved properties compared to bare copper, this layer itself does not melt during soldering but dissolves in the solder and reduces wettability of the solder, and thus the soldered samples break more quickly on the silver surface than on bare copper [26,27]. However, immersion silver was more suitable for ICA bonding than bare copper. One reason for this could be that the corrosion potential between the ICAs on bare copper surface finish leads to the deterioration of the adhesive bonding. However, since the ICAs contain silver particles, this corrosion potential is eliminated via adhesive bonding using ICAs on silver surface [25].

Another factor that plays an important role in the reliability of the soldered samples could be the soldering temperature. At increased soldering temperatures, the electronic components and circuit carriers are exposed to increased thermal stress. As a result, defects can occur. In general, defects such as delamination can also occur more frequently at high process temperatures. For this reason, the soldered samples with increased process temperature can fail earlier than the samples with low soldering temperature [28,29].

Solder A has poor wetting properties compared to the other solder materials. In addition, the soldering process takes place at a higher temperature, which can lead to damage of the components. Solder C has very good wetting properties compared to the other solders, and is processed at a lower temperature, which has a positive effect on the entire process [30].

The failure mechanisms are different for soldered and adhesively bonded components. If we look at the SEM images in the Figure 13, we see that the soldered samples tend to break more at the solder/trace junction, while the adhesively bonded components tend to break in the middle of the adhesive.

## 5. Conclusions

The following points can be summarized and highlighted from this study:

- Based on shear strength tests, it can be concluded that the choice of the conductive adhesive is the most important factor for ensuring that the mechanical connection is maintained. The variation of process parameters tends to play a minor role.
- Soldering enables higher shear strength values than conductive adhesive bonding.
- In the dynamic bending test, more samples endured to the end of the test of 50,000 cycles in the conductive adhesive bonding group than in the soldering group.
- In the soldering group, solder C on bare copper surface achieved more cycles to failure than the other solders.
- In the conductive adhesive bonding group, adhesive H20E on immersion silver surface finish achieved more cycles to failure than the other adhesive.
- The bare copper surface is more suitable than the immersion silver surface finish for reliable solderability.
- The immersion silver surface finish is better than the bare copper surface for reliable conductive adhesive bonding.

## Figures and Tables

**Table 1:** Factors and levels for DoE of shear tests for soldering and conductive adhesive bonding. The factors and levels are evaluated in a full factorial manner, i.e., all factors are examined with all possible combinations.

Factors	Level 1	Level 2	Level 3
Component size	0603	0402	-
Solder type	SAC (solder A)	SnBi (solder B)	SnBiAg (solder C)
Soldering process	Continuous furnace	Vapor phase (just for solder A)	-
Conductive adhesive	Adhesive A	Adhesive B	-
Adhesive reservoir depth	150 µm	100 µm	-
Curing profile	150 °C/12 min	125 °C/30 min	-

**Table 2:** Factors and levels for DoE of dynamic bending tests for soldering and conductive adhesive bonding. The factors and levels are evaluated in a full factorial manner, i.e., all factors are examined with all possible combinations.

Factors	Level 1	Level 2	Level 3
Component size	0603	0402	-
Final metal surface on substrate	Bare copper	Immersion silver	
Solder type	SAC (solder A)	SnBi (solder B)	SnBiAg (solder C)
Conductive adhesive	Adhesive A	Adhesive B	-

## Data Availability

Not applicable.
